# Hyperuricemia and dementia – a case-control study

**DOI:** 10.1186/s12883-018-1136-y

**Published:** 2018-08-31

**Authors:** Bettina Engel, Willy Gomm, Karl Broich, Wolfgang Maier, Klaus Weckbecker, Britta Haenisch

**Affiliations:** 1 Institute of General Practice and Family Medicine, University of Bonn, Bonn, Germany; 2 Department of Health Services Research, Division of General Medicine, University of Oldenburg, Oldenburg, Germany; 3 German Center for Neurodegenerative Diseases (DZNE), Bonn, Germany; 4 Federal Institute for Drugs and Medical Devices (BfArM), Kurt-Georg-Kiesinger-Allee 3, D-53175 Bonn, Germany; 5 Department of Psychiatry, University of Bonn, Bonn, Germany; 6 Center for Translational Medicine, University of Bonn, Bonn, Germany

**Keywords:** Epidemiology, Gout, Hyperuricemia, Treatment, Dementia

## Abstract

**Background:**

There is evidence that uric acid may have antioxidant and neuroprotective effects and might therefore alter the risk for neurodegenerative diseases such as dementia. So far, the relation between serum uric acid (SUA) levels or hyperuricemia and dementia remains elusive. Most studies focused on the disease or SUA levels. Effects of anti-hyperuricemic treatment have not been considered yet. This study investigated the association between hyperuricemia and dementia taking into account anti-hyperuricemic treatment.

**Methods:**

We used longitudinal German public health insurance data and analyzed the association between hyperuricemia with and without different treatment options and dementia in a case-control design. Applying logistic regression the analysis was adjusted for several potential confounders including various comorbidities and polypharmacy.

**Results:**

We identified 27,528 cases and 110,112 matched controls of which 22% had a diagnosis of hyperuricemia or gout and 17% received anti-hyperuricemic drugs. For patients with a diagnosis of hyperuricemia we found a slightly reduced risk for dementia (adjusted odds ratio [OR] 0.94, 95% confidence interval [CI] 0.89 to 0.98). The risk reduction was more pronounced for patients treated with anti-hyperuricemic drugs (adjusted OR 0.89, 95% CI 0.85 to 0.94, for regular treatment).

**Conclusions:**

Our results showed a slight reduction for dementia risk in patients with hyperuricemia, both with and without anti-hyperuricemic treatment.

## Background

Hyperuricemia and dementia are both well-known and common diseases. Alzheimer’s disease (AD) is the most common cause of dementia; vascular dementia is very common in older individuals with dementia, often occurring in mixed dementia pathologies together with AD [1]. Although the exact biological mechanisms by which neuronal damage in dementia takes place are still not fully understood, there are risk factors that have been shown to potentially alter dementia risk [1]. Stroke and metabolic syndrome have been detected as risk factors for vascular and all-cause dementia [2, 3]. Gout and hyperuricemia have previously been found to be associated with metabolic syndrome and cardiovascular diseases [4, 5]. Therefore, patients with gout or hyperuricemia might have a modified- maybe higher- risk of dementia. On the contrary, uric acid has antioxidant properties and could therefore exert potentially neuroprotective effects [6, 7]. Evidence suggests that gout is associated with a lower risk for Parkinson’s disease (PD) [8–10]. The exact underlying mechanism by which uric acid protects against PD is still unclear, but it has been hypothesized that uric acid may ameliorate oxidative stress, a pathogenic pathway in PD [11], as well as in other neurodegenerative diseases such as dementia [12]. Indeed, previous studies detected that serum uric acid (SUA) levels in patients with mild cognitive impairment or AD are lower compared to healthy controls [13, 14]. However, studies on the association of SUA levels with cognitive decline or dementia are conflicting [15]. A cohort study by Euser et al. revealed that higher SUA levels are associated with a better cognitive function in late life and a decreased risk of dementia [16]. A recent study by Latourte et al. detected a higher risk for AD and vascular or mixed dementia with high SUA levels [17]. However, Latourte et al. excluded patients receiving urate-lowering treatments so they calculated the association within normal SUA level ranges while Euser et al. did not exclude gout or hyperuricemia patients. Another limitation of both studies is that SUA level was assessed only at baseline and up to 12 years before dementia was diagnosed. Furthermore, the sample size of cohort data studies is generally small, limiting the generalizability of the results. Currently, only two studies examined the connection between gout or hyperuricemia and dementia on a population-based level: one study involved claims data from Taiwan, examining the effect on vascular and non-vascular dementia, the other one is based on computerized medical records from general practitioners in the UK and was restricted to Alzheimer’s disease as the outcome parameter [18, 19]. None of the previous studies included the effects of different anti-hyperuricemic treatment. In the present study, we use a large longitudinal German claims dataset and apply a matched case-control design to evaluate the effect of hyperuricemia on the risk of incident any dementia. To increase generalizability and assess an unselected patient population we include patients who did or did not receive anti-hyperuricemic drugs. We further differentiate between occasional and intensive treatment with anti-hyperuricemic drugs.

## Methods

### Data source and study design

A longitudinal sample of the largest German statutory health insurance, Allgemeine Ortskrankenkasse (AOK), was used to conduct case-control analyses. The AOK covers about 50% of the German population at least 80 years old and one third of the total population at least 50 years old [20]. The AOK data set includes the ambulatory as well as the hospital sector of the health care system. The ambulatory sector is an important source of medical care as it consists of a network of ambulatory primary and specialist care professionals.

The data include information on basic demographics like age and gender, as well as inpatient and outpatient diagnoses (coded by the International Classification of Diseases-10, ICD-10) and filled drug prescriptions (categorized according to the Anatomical-Therapeutic-Chemical classification system, ATC code). Data are compiled on a quarterly basis for the years 2004–2013. As a baseline interval where initially no dementia was claimed we used the year 2004 in the data set. A lag time of two years before the first valid diagnosis of dementia was introduced. The index date is the first valid dementia diagnosis. The observation time is defined from study start until begin of the lag time before index date. The minimal follow-up time is three years (one year minimal observation time plus two years lag time).

### Dementia diagnosis and case-control matching

Patients aged 60 years or older were included. Cases were defined as patients who had a dementia diagnosis in at least 75% of all quarters after the first valid diagnosis within the study period and had no data inconsistencies (missing birthdate, date of death before start of study, different gender in different years). The following ICD-10 codes for dementia were used: G30 (Alzheimer’s disease), F00 (Dementia in Alzheimer’s disease), F01 (Vascular dementia), F02 (Dementia in other diseases), F03 (Unspecified dementia), F05.1 (Delirium superimposed on dementia), G31.1 (Senile degeneration of brain), G31.82 (Lewy bodies dementia), and G31.9 (Degenerative disease of nervous system, unspecified). We considered diagnoses as valid if they were hospital diagnoses or reported as verified by the physician for the outpatient sector. Patients were excluded if they had less than three years of follow-up in the study, if they had any dementia diagnosis in the baseline interval (year 2004), or if they had less than 75% of all quarters after the index quarter with a valid dementia diagnosis. Cases were matched to four controls without replacement on age (± one year) at study begin and gender. Matched cases and controls have the same study begin and index date; thus, all patients have the same follow-up time in each match group.

### Hyperuricemia/gout diagnosis

Cases and controls were categorized into six groups according to hyperuricemia/ gout diagnoses (ICD-10: E79, M10, M11.8, M11.9) and use of anti-hyperuricemic drugs (ATC codes: M04AA01, M04AA02, M04AA03, M04AA51, M04AB01, M04AB02, M04AB03, M04AB04, M04AX01, M04AX02, V03AF07). As the aim of our study was to show the correlation between hyperuricemia and dementia, we did not differentiate gout and hyperuricemia. The diagnosis gout implicates hyperuricemia. Drug use was separated into two categories: occasional use defined as one to three quarters, and intensive use: four and more quarters with prescriptions in the observation time. Combining the status of hyperuricemia diagnosis and drug use led to the following six groups. The reference category was no diagnosis and no drug use. The exposed groups were: no diagnosis with occasional drug use, no diagnosis with intensive drug use, diagnosis but no drug use, diagnosis with occasional drug use, and diagnosis with intensive drug use.

### Statistical analyses

We adjusted the analysis for the following potential confounders: age, gender, polypharmacy (defined as five or more drug prescriptions besides anti-hyperuricemic drugs) and the comorbidities depression (ICD-10: F32-F34, F38, F39), stroke (ICD-10: I63, I64, I69.3, I69.4, G45), ischemic heart disease (ICD-10: I20-I25), other cerebrovascular diseases (ICD-10: I65-I67,I69.8), diabetes (ICD-10: E10-E14, E89.1), polyarthritis (ICD-10: M05-M09), atherosclerosis (ICD-10: I70), hypertension (ICD-10: I10-I13, I15), renal impairment (ICD-10: N18, N19) and hyperlipidemia (ICD-10: E78.0-E78.5, E78.8, E78.9). The covariates were selected based on existing evidence and previous publications on the topic of dementia risk and gout diagnoses [18, 20–22]. We considered comorbidity as present if it was reported in at least one quarter during the observation time, and in at least two quarters during the study time.

We examined the effect of hyperuricemia diagnosis and drug use status on incident any dementia using a multinomial variable including the following values 0: reference category with no diagnosis and no drug use, 1: no diagnosis with occasional drug use, 2: no diagnosis with intensive drug use, 3: diagnosis without any drug use, 4: diagnosis with occasional drug use, 5: diagnosis with intensive drug use. Conditional logistic regression was applied. The match groups were used as strata. The dependent variable was the occurrence of incident any dementia. The analysis was adjusted for potential confounding factors as described above. We applied backward selection to remove variables with non-significant effects on the outcome. All calculations were done using SAS 9.3 for Windows. We considered *p* < 0.05 (two tailed) to be statistically significant.

## Results

### Sample characteristics

**Fig. 1:**
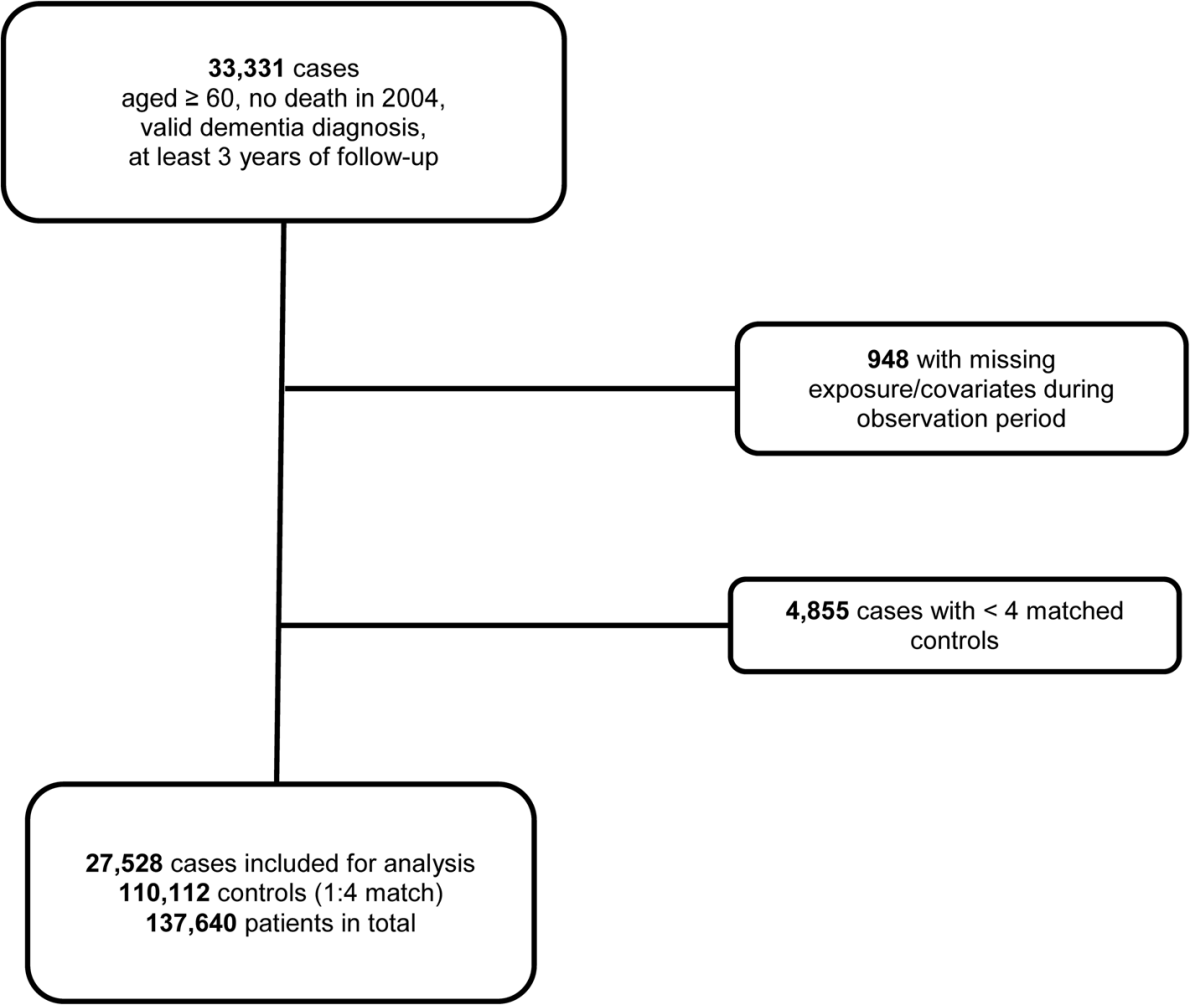
Sample for analyses

We identified 33,331 persons aged 60 years or older at the beginning of the study period in 2004 with no dementia at baseline, a valid dementia diagnosis afterwards, and at least three years of follow-up. Of these, 5803 were excluded after filtering for quality control criteria (see Fig. 1). In total, we included 137,640 patients, 27,528 cases and 110,112 controls in our study (Table 1). The overall mean age of these patients in 2004 was 73.9 (±6.5) years; 63% were female, 37% were male patients (Table 1).The mean age at first dementia diagnosis was 80.9 (±6.3) years.

**Table 1:** Descriptive results

	*N* (% from 137,640)	Cases (% from 27,528)	Controls (% from 110,112)
Female	86,910 (63.1)	17,382 (63.1)	69,528 (63.1)
Male	50,730 (36.9)	10,146 (36.9)	40,584 (36.9)
Mean age in 2004 (SD)	73.9 (6.5)	74.0 (6.5)	73.9 (6.5)

### Anti-hyperuricemic drugs

In our sample, we detected 23,370 patients with a prescription of anti-hyperuricemic drugs. Allopurinol was by far the most frequently prescribed drug (98.4%), followed by Benzbromarone (1.8%), Allopurinol combinations (1.3%), Febuxostat (0.2%), Probenecid (0.06%), and Rasburicase (0.01%; including multiple different prescriptions at a time). We analyzed the daily prescribed dose of Allopurinol (DDD 0.4 g/d, 90d/quarter). 35.5% of patients received 300 mg/d, another 35.7% less than 200 mg/d, 12.1% 200–300 mg/d, and 16.8% received more than 300 mg/d allopurinol (see Table 2).

**Table 2:** Dose categories for allopurinol prescriptions

Daily Dose Allopurinol (mg) (DDD 0.4 g/d, 90d/quarter)	%
< 200	35.7
200–300	12.1
300	35.5
300–400	12.7
400–600	3.7
> 600	0.4

### Association between hyperuricemia and dementia

After grouping the cases in six categories according to hyperuricemia/gout diagnosis and use of any anti-hyperuricemic drugs as described in material and methods we found 2379 patients (8.6% of all cases; Table 3) with the diagnosis of any type dementia and the diagnosis of hyperuricemia or gout as well as at least four quarters with a prescription of anti-hyperuricemic drugs (intensive drug use). 1168 patients (4.2% of all cases; Table 3) had the diagnosis of hyperuricemia or gout, but received anti-hyperuricemic drugs in less than four quarters (occasional drug use). 2590 patients (9.4% of all cases; Table 3) had the diagnosis of hyperuricemia or gout but received no anti-hyperuricemic drugs. Another group included patients without a diagnosis of hyperuricemia or gout and intensive (*n* = 558; Table 3) or occasional (*n* = 629; Table 3) anti-hyperuricemic drug use. The category of patients with neither diagnosis of hyperuricemia or gout nor anti-hyperuricemic therapy (*n* = 20,204, 73.4% of all cases; Table 3) represented the cases within our reference group.

**Table 3:** Association between gout or hyperuricemia and dementia, different treatment/diagnosis groups

Diagnosis (hyperuricemia or gout), Treatment regimen (antihyperuricemic drug use)^a^	Controls (*n*=110,112) (% from 137,640)	Cases (*n*=27,528) (% from 137,640)	OR (95% CI), adjusted	*p*-value, adjusted for covariates
D:0, T:0	81,091 (58.9)	20,204 (14.7)	Ref	
D:0, T:1 < =qu < 4	2445 (1.8)	629 (0.5)	0.93 (0.85,1.02)	0.11
D:0, T:qu > =4	2084 (1.5)	558 (0.4)	0.95 (0.86,1.04)	0.26
D:1, T:0	10,385 (7.6)	2590 (1.9)	0.94 (0.89,0.98)	0.0065
D:1, T:1 < =qu < 4	4718 (3.4)	1168 (0.9)	0.89 (0.83,0.95)	< 0.001
D:1, T:qu > =4	9389 (6.8)	2379 (1.7)	0.89 (0.85,0.94)	< 0.001

Global test: Wald Χ^2^ = 2403.13, DF = 14, *p* < 0.001

^a^Treatment: T:0 means no anti-hyperuricemic treatment, qu > =x: means x or more quarters with treatment in the observation period, D:0 means no hyperuricamia diagnosis

Patients with a diagnosis of hyperuricemia or gout (D:1) without and with anti-hyperuricemic therapy have a slight, but significant reduced risk for incident any dementia. This finding is consistent for no drug use (OR 0.94 [CI 0.89–0.98]; Table 2), for occasional (T:1 < =qu < 4: OR 0.89 [CI 0.83–0.95]; Table 2) as well as intensive anti-hyperuricemic drug use (T:qu ≥ 4: OR 0.89 [0.85–0.94]; Table 2). Patients with no diagnosis of hyperuricemia or gout, but with anti-hyperuricemic drug prescription showed no significant risk reduction, neither for occasional (OR 0.93 [0.85–1.02]) nor for intensive anti-hyperuricemic drug use (OR 0.95 [0.86–1.04], Table 2).

Our analysis was adjusted for potential confounders as shown in Table 4. Of the included covariates, we detected the highest risk increase for incident any dementia with stroke, depression, cerebrovascular diseases, and diabetes (OR 1.53 [CI 1.47–1.59], OR 1.50 [CI 1.46–1.55], OR 1.32 [CI 1.27–1.37], and OR 1.29 [CI 1.25–1.33], respectively; Table 4). Polypharmacy (OR 1.15 [CI 1.11–1.19]) and renal impairment (OR 1.11 [CI 1.06–1.15]) also increased dementia risk (see Table 4). The use of anti-hyperuricemic drugs (OR 0.94 [CI 0.90–0.99]), hyperuricemia (OR 0.94 [CI 0.90–0.98], hyperlipidemia (OR 0.87 [CI 0.84–0.89]), and hypertension (OR 0.92 [CI 0.89–0.96]) slightly decreased dementia risk (see Table 3). Two covariates (atherosclerosis and polyarthritis) were removed by backward selection with *p* ≥ 0.05.

**Table 4:** Association between gout/hyperuricemia and dementia including covariates, all patients, no selection according to treatment

Covariate	N (% from 137,640)	Cases (% from 27,528)	Controls (% from 110,112)	OR (CI)	*p*-value
Anti-hyperuricemia drugs	23,370 (17.0)	4.734 (17.2)	18,636 (16.9)	0.94 (0.90,0.99)	0.01
Hyperuricemia	30,629 (22.3)	6137 (22.3)	24,492 (22.2)	0.94 (0.90,0.98)	0.0025
Polypharmacy^a^	90,418 (65.7)	19,495 (70.8)	70,923 (64.4)	1.15 (1.11,1.19)	< 0.001
Diabetes	48,553 (35.3)	11,247 (40.9)	37,306 (33.9)	1.29 (1.25,1.33)	< 0.001
Ischemic heart disease	55,307 (40.2)	11,862 (43.1)	43,445 (39.5)	1.04 (1.01,1.07)	0.005
Stroke	16,429 (11.9)	4716 (17.1)	11,713 (10.6)	1.53 (1.47,1.59)	< 0.001
Other cerebrovascular diseases	21,754 (15.8)	5670 (20.6)	16,084 (14.6)	1.32 (1.27,1.37)	< 0.001
Atherosclerosis	17,339 (12.6)	3898 (14.2)	13,441 (12.2)	Removed from model	0.078
Hypertension	113,640 (82.6)	22.978 (83.5)	90,662 (82.3)	0.92 (0.89,0.96)	< 0.001
Renal impairment	18,769 (13.6)	4285 (15.6)	14,484 (13.2)	1.11 (1.06,1.15)	< 0.001
Hyperlipidemia	70,373 (51.1)	13,877 (50.4)	56,496 (51.3)	0.87 (0.84,0.89)	< 0.001
Depression	27,567 (20.0)	7257 (26.4)	20,310 (18.4)	1.50 (1.46,1.55)	< 0.001
Polyarthritis	5692 (4.1)	1196 (4.3)	4496 (4.1)	Removed from model	0.73

Global test: Wald Χ^2^ = 2402.99, DF = 11, *p* < 0.001

^a^Polypharmacy means prescription of 5 or more different drugs (ATC codes) besides anti-hyperuricemic drugs in a quarter

## Discussion

Our results showed a slight reduction for dementia risk in patients with a diagnosis of hyperuricemia or gout and occasional or intensive anti-hyperuricemic treatment. This category of patients is supposed to have the highest uric acid levels because the disease requires treatment. Similarly, patients with a hyperuricemia or gout diagnosis, but without treatment also displayed a reduced risk for dementia. The groups of patients without a particular hyperuricemia or gout diagnosis, but with occasional or intensive anti-hyperuricemic drug prescription, showed no significantly reduced dementia risk.

In theory, the prescription of anti-hyperuricemic drugs should correlate with the diagnosis of gout or hyperuricemia. Giersiepen et al. used German statutory health insurance data and showed a correlation of anti-hyperuricemic drug prescription in 27.7% of patients with gout diagnosis and in a further 16.2% of patients with hyperuricemia after three years of prescription of anti-hyperuricemic drugs [23]. This displays a considerable amount of under-documentation of hyperuricemia or gout in Germany, similar to other countries [24, 25].

Uric acid is the pathogenic factor for the development of gout. Hyperuricemia can lead to gout that is characterized by deposition of urate crystals, mostly in joints, connective tissue and kidneys. The aim of gout treatment is to reduce the uric acid level (below the solubility product of 6.5 mg/dl) [26]. Different anti-hyperuricemic drugs are available. The most common drug used is Allopurinol [27]. Our study showed that Allopurinol accounts for 98% of all anti-hyperuricemic drug prescriptions. Further anti-hyperuricemic drug prescriptions are for Benzbromarone, Febuxostat, Rasburicase and Probenecid as described in the results. In our study we calculated that 47.8% of patients received less than 300 mg/d Allopurinol, and 35.5% received 300 mg/d. As described in other studies, about 300 mg/d of the anti-hyperuricemic drug Allopurinol is needed to reach the SUA target level [28, 29]. However, some studies showed that patients requiring anti-hyperuricemic treatment often receive insufficient dosage of anti-hyperuricemic drugs (e.g. < 300 mg/d Allopurinol) [28, 29]. This suggests that a large proportion of patients requiring treatment in our study presumably displays SUA levels which are above the SUA target level. A main reason for insufficient therapy can be that regular SUA level control after treatment initiation is neglected [30]. Hence, no or insufficient dose adjustment takes place. Thus, the effect of a slightly reduced dementia risk in patients with anti-hyperuricemic drug treatment could be interpreted as a result from still elevated SUA levels in these patients. We did not find evidence that anti-hyperuricemic drug treatment itself has a significant modifying effect on dementia risk.

Exact biological mechanisms by which SUA levels might contribute to the observed inverse association with dementia risk are yet to be explored. The frequently discussed hypothesis includes that uric acid has antioxidative properties and might be able to reduce oxidative stress by being a scavenger of biological oxidants such as peroxynitrite radicals which have been shown to be involved in the pathology of neurodegenerative diseases [31]. In this way, uric acid exerts neuroprotective effects by ameliorating free-radical-induced protein and DNA damage [32]. Furthermore, uric acid has been shown to act as an electron donor that increases antioxidant enzyme activity (e.g. superoxide dismutase) [33]. The brain is especially susceptible to oxidative stress and a dysfunction of antioxidative properties has been reported to contribute to neurodegenerative diseases [34].

Our results are in line with other claims data studies. A Taiwanese study with national health insurance data also showed that patients with gout have a lower risk for incident dementia (HR 0.77 CI 0.72–0.82, for all gout patients in the adjusted model) [18]. Lu et al. used medical record data from general practitioners in the UK and detected an inverse association between gout and the risk of developing AD, supporting the potential neuroprotective role of uric acid [19]. The authors observed an hazard ratio (HR) of 0.76 (CI 0.66–0.87) for AD risk with gout in the adjusted model [19]. However, our results are slightly less pronounced.

Our findings do not support the results by Latourte et al. who reported elevated risks for dementia with higher SUA levels [17]. This might be due to differences in study populations. Latourte et al. analyzed the effect of different SUA levels mostly within the normal, not elevated range and excluded patients receiving urate-lowering medication [17]. It is therefore difficult to judge if further confounding factors that were not addressed in their study might have contributed to the effect. Furthermore, the sample size was limited, including only 110 all-cause dementia cases, leading to non-significant effects for most SUA level categories [17]. In a sensitivity analysis with an usual hyperuricemia threshold the effect was not significant [17]. Time-varying effects were not taken into account as SUA levels were based on a single measurement, up to 12 years prior dementia diagnosis [17].

Our study has several strengths. For our study we included a large data set of treated and untreated hyperuricemia or gout patients and controls. The sample is population-based and covers longitudinal data from 2004 to 2013 extracted from the largest German statutory public health insurance. This allowed us to perform the analysis in an unselected patient population. Health claims data cover the total population, not only community-dwelling individuals. The sample also includes people who are excluded in most cohort studies, namely persons who live in institutions such as assisted living or nursing homes. Furthermore, with the use of routine database records selection bias or recall bias is avoided.

There are also limitations. Because we make use of claims data we cannot completely rule out residual confounding. However, we adjusted our analysis by including potential confounding factors such as polypharmacy and comorbidities. Because we analyzed claims data with a high number of diagnoses of unspecified and mixed dementia, we were not able to differentiate between different dementia etiologies, such as dementia in the course of AD or vascular dementia. This is why we do not perform subgroup analyses for different dementia types. In addition, claims data lack data on SUA levels. Thus, we rely on prescription data and are not able to confirm SUA level ranges of treated or untreated patients.

## Conclusion

Using German claims data our study showed a slight reduction for dementia risk in patients with hyperuricemia or gout diagnosis and occasional as well as regular anti-hyperuricemic treatment. Patients without targeted treatment also displayed a decreased risk for dementia. Our finding confirms previous studies with medical record and claims data from the UK and Taiwan that hyperuricemia or gout is inversely associated with dementia risk. More research is needed to gain more evidence of a potential neuroprotective mechanism of high SUA levels.

## Abbreviations

AD: Alzheimer’s disease
AOK: Allgemeine Ortskrankenkasse
ATC: Anatomical-Therapeutic-Chemical
CI: Confidence interval
DDD: Defined daily dose
ICD-10: International Classification of Diseases, tenth revision
OR: Odds ratio
PD: Parkinson’s disease
SUA: Serum uric acid
